# Dibromido[(1*R*,2*R*,*N* ^1^ *S*)-*N*-(pyridin-2-ylmeth­yl)cyclo­hexane-1,2-diamine-κ^3^ *N*,*N*′,*N*′′]cadmium

**DOI:** 10.1107/S1600536811007033

**Published:** 2011-03-02

**Authors:** Run-Ting Yin, Zhe Cao, Lin Cheng

**Affiliations:** a Department of Chemistry and Chemical Engineering, Southeast University, Nanjing 211189, People’s Republic of China

## Abstract

In the title compound, [CdBr_2_(C_12_H_19_N_3_)], the Cd^II^ atom is coordinated by the three N atoms of the (1*R*,2*R*)-*N*-(pyridin-2-ylmeth­yl)cyclo­hexane-1,2-diamine ligand and a bromide ion in the basal plane, and by a second bromide in the apical position. The coordination environment can be described as distorted square pyramidal. The coordination of the enanti­o­pure ligand to the metal atom renders the central N atom chiral with an *S* configuration, so the complex is enanti­omerically pure and corresponds to the *S*,*R*,*R* diastereoisomer. In the crystal, the mol­ecules are linked *via* weak N—H⋯Br hydrogen bonds into a chain parallel to the *b* axis.

## Related literature

For related structures, see: Gou *et al.* (2010 ▶). For non-linear optical properties of chiral coordination polymers, see: He *et al.* (2010 ▶).
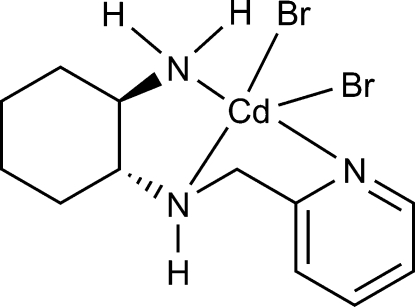


## Experimental

### 

#### Crystal data

[CdBr_2_(C_12_H_19_N_3_)]
*M* *_r_* = 477.52
Orthorhombic, 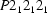

*a* = 8.7153 (16) Å
*b* = 9.1978 (17) Å
*c* = 19.759 (4) Å
*V* = 1583.9 (5) Å^3^
*Z* = 4
Mo *K*α radiation
μ = 6.41 mm^−1^
*T* = 173 K
0.12 × 0.09 × 0.08 mm

#### Data collection

Bruker SMART APEX CCD diffractometer
Absorption correction: multi-scan (*SADABS*; Sheldrick, 2008 ▶) *T*_min_ = 0.513, *T*_max_ = 0.628
9389 measured reflections
3048 independent reflections
2348 reflections with *I* > 2σ(*I*)
*R*_int_ = 0.048

#### Refinement

*R*[*F* ^2^ > 2σ(*F* ^2^)] = 0.033
*wR*(*F* ^2^) = 0.052
*S* = 0.96
3048 reflections
163 parameters
H-atom parameters constrained
Δρ_max_ = 0.49 e Å^−3^
Δρ_min_ = −0.48 e Å^−3^
Absolute structure: Flack (1983 ▶), 1244 Friedel pairs
Flack parameter: 0.049 (11)

### 

Data collection: *SMART* (Bruker, 2000 ▶); cell refinement: *SAINT-Plus* (Bruker, 2000 ▶); data reduction: *SAINT-Plus*; program(s) used to solve structure: *SHELXTL* (Sheldrick, 2008 ▶); program(s) used to refine structure: *SHELXL97* (Sheldrick, 2008 ▶); molecular graphics: *ORTEPIII* (Burnett & Johnson, 1996 ▶), *ORTEP-3 for Windows* (Farrugia, 1997 ▶) and *PLATON* (Spek, 2009 ▶); software used to prepare material for publication: *SHELXTL*.

## Supplementary Material

Crystal structure: contains datablocks I, global. DOI: 10.1107/S1600536811007033/dn2658sup1.cif

Structure factors: contains datablocks I. DOI: 10.1107/S1600536811007033/dn2658Isup2.hkl

Additional supplementary materials:  crystallographic information; 3D view; checkCIF report

## Figures and Tables

**Table 1:** Hydrogen-bond geometry (Å, °)

*D*—H⋯*A*	*D*—H	H⋯*A*	*D*⋯*A*	*D*—H⋯*A*
N3—H3*B*⋯Br2^i^	0.92	2.89	3.750 (5)	156
N3—H3*C*⋯Br1^ii^	0.92	2.59	3.498 (5)	168

Symmetry codes: (i) 
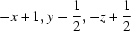
; (ii) 
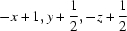
.

**Fig. 1.**
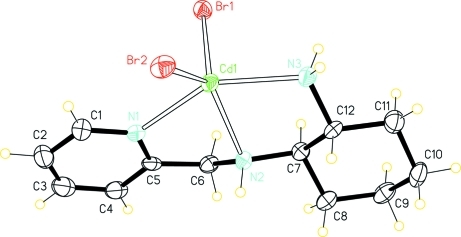
View of the title compound with the atom-labelling scheme. Displacement ellipsoids are drawn at the 30% probability level. H atoms are represented as small spheres of arbitrary radii.

**Fig. 2.**
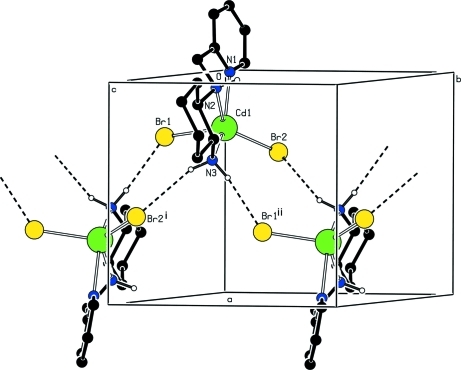
Partial packing view showing the chain developping parallel to the b axis. H atoms not involved in hydrogen bondings have been omitted for clarity. H bonds are shown as dashed lines. [Symmetry codes: (i) -x+1, y-1/2, -z+1/2; (ii) -x+1, y+1/2, -z+1/2]

## supplementary crystallographic information

### Comment

Recently, rational design and synthesis of chiral coordination polymers have been of great interests due to their potential utility in enantiomerically selective catalysis and separations, second-order nonlinearoptical (NLO) applications and luminescence (He *et al.*, 2010). A simple and effective design route for such polymers is to appropriately organize the metal ions into ordered architectures by use of chiral ligands. Herein, we report a new chiral complex, Zn(pcd)Br_2_ (pcd = (1*R*,2*R*)-*N*^1^-(pyridin-2-ylmethyl)cyclohexane -1,2-diamine), with a enantiomerically pure pcd ligand.

The title compound is a mononuclear complex, in which the coordination environment of Cd^II^ ion can be described as distorted square-pyramidal, being surrounded by one tridentate ligand and two bromine anions (Fig. 1).

The molecules are linked to each other, *via* weak N—H···Br hydrogen bonds, into a one-dimensional hydrogen bonding network developping parallel to the b axis (Table 1, Fig. 2).

### Experimental

(1*R*,2*R*)-*N*^1^-(pyridin-2-ylmethyl)cyclohexane -1,2-diamine (0.041 g, 0.2 mmol) dissolved in water (8 ml) was added to a methanol solution (10 ml) of CdBr_2_ (0.054 g, 0.2 mmol). The mixture solution was stirred for 1 h at room temperature and then filtered. The filtrate was allowed to evaporate slowly at room temperature. After 2 weeks, yellow block crystals were obtained in 32.5% yield (0.031 g).

### Refinement

All H atoms were fixed geometrically and treated as riding with C—H = 0.93–0.97 Å and N—H = 0.92–0.93 Å with *U*_iso_(H) = 1.2 *U*_eq_(C or N) and *U*_iso_(H) = 1.5 *U*_eq_(O).

### Crystal data

**Table:**

[CdBr_2_(C_12_H_19_N_3_)]	*F*(000) = 920
*M**_r_* = 477.52	*D*_x_ = 2.003 Mg m^−^^3^
Orthorhombic, *P*2_1_2_1_2_1_	Mo *K*α radiation, λ = 0.71073 Å
Hall symbol: P 2ac 2ab	Cell parameters from 792 reflections
*a* = 8.7153 (16) Å	θ = 2.5–28.0°
*b* = 9.1978 (17) Å	µ = 6.41 mm^−^^1^
*c* = 19.759 (4) Å	*T* = 173 K
*V* = 1583.9 (5) Å^3^	Block, yellow
*Z* = 4	0.12 × 0.09 × 0.08 mm

### Data collection

**Table:**

Bruker SMART APEX CCD diffractometer	3048 independent reflections
Radiation source: fine-focus sealed tube	2348 reflections with *I* > 2σ(*I*)
graphite	*R*_int_ = 0.048
φ and ω scans	θ_max_ = 26.0°, θ_min_ = 2.4°
Absorption correction: multi-scan (*SADABS*; Sheldrick, 2008)	*h* = −10→9
*T*_min_ = 0.513, *T*_max_ = 0.628	*k* = −11→10
9389 measured reflections	*l* = −23→22

### Refinement

**Table:**

Refinement on *F*^2^	Secondary atom site location: difference Fourier map
Least-squares matrix: full	Hydrogen site location: inferred from neighbouring sites
*R*[*F*^2^ > 2σ(*F*^2^)] = 0.033	H-atom parameters constrained
*wR*(*F*^2^) = 0.052	*w* = 1/[σ^2^(*F*_o_^2^) + (0.0014*P*)^2^ + 0.1*P*] where *P* = (*F*_o_^2^ + 2*F*_c_^2^)/3
*S* = 0.96	(Δ/σ)_max_ < 0.001
3048 reflections	Δρ_max_ = 0.49 e Å^−^^3^
163 parameters	Δρ_min_ = −0.47 e Å^−^^3^
0 restraints	Absolute structure: Flack (1983), 1244 Friedel pairs
Primary atom site location: structure-invariant direct methods	Flack parameter: 0.049 (11)

### Special details

**Table:**

Geometry. All e.s.d.'s (except the e.s.d. in the dihedral angle between two l.s. planes) are estimated using the full covariance matrix. The cell e.s.d.'s are taken into account individually in the estimation of e.s.d.'s in distances, angles and torsion angles; correlations between e.s.d.'s in cell parameters are only used when they are defined by crystal symmetry. An approximate (isotropic) treatment of cell e.s.d.'s is used for estimating e.s.d.'s involving l.s. planes.
Refinement. Refinement of *F*^2^ against ALL reflections. The weighted *R*-factor *wR* and goodness of fit *S* are based on *F*^2^, conventional *R*-factors *R* are based on *F*, with *F* set to zero for negative *F*^2^. The threshold expression of *F*^2^ > σ(*F*^2^) is used only for calculating *R*-factors(gt) *etc*. and is not relevant to the choice of reflections for refinement. *R*-factors based on *F*^2^ are statistically about twice as large as those based on *F*, and *R*- factors based on ALL data will be even larger.

### Fractional atomic coordinates and isotropic or equivalent isotropic displacement parameters (Å^2^)

**Table:**

	*x*	*y*	*z*	*U*_iso_*/*U*_eq_	
Cd1	0.24783 (5)	0.10173 (5)	0.207493 (18)	0.03280 (11)	
Br1	0.29207 (7)	−0.17278 (7)	0.18146 (3)	0.04158 (18)	
Br2	0.35856 (7)	0.29959 (7)	0.13039 (3)	0.04411 (18)	
C1	−0.0384 (8)	0.1320 (7)	0.0949 (3)	0.052 (2)	
H1A	0.0404	0.1650	0.0654	0.062*	
C2	−0.1845 (8)	0.1196 (8)	0.0697 (3)	0.062 (2)	
H2A	−0.2071	0.1427	0.0239	0.075*	
C3	−0.2968 (8)	0.0725 (8)	0.1131 (4)	0.065 (2)	
H3A	−0.3993	0.0630	0.0973	0.077*	
C4	−0.2620 (8)	0.0390 (7)	0.1792 (3)	0.0499 (16)	
H4A	−0.3396	0.0064	0.2094	0.060*	
C5	−0.1119 (7)	0.0538 (6)	0.2009 (3)	0.0353 (15)	
C6	−0.0630 (6)	0.0155 (7)	0.2716 (3)	0.0373 (16)	
H6A	−0.1516	0.0257	0.3026	0.045*	
H6B	−0.0290	−0.0872	0.2728	0.045*	
C7	0.1349 (6)	0.0623 (6)	0.3592 (3)	0.0336 (15)	
H7A	0.1528	−0.0449	0.3564	0.040*	
C8	0.0343 (7)	0.0913 (8)	0.4213 (3)	0.0448 (18)	
H8A	0.0029	0.1947	0.4214	0.054*	
H8B	−0.0599	0.0314	0.4181	0.054*	
C9	0.1152 (7)	0.0572 (7)	0.4877 (3)	0.0508 (19)	
H9A	0.0488	0.0868	0.5260	0.061*	
H9B	0.1323	−0.0490	0.4911	0.061*	
C10	0.2669 (8)	0.1347 (7)	0.4929 (3)	0.0550 (19)	
H10A	0.3191	0.1056	0.5353	0.066*	
H10B	0.2492	0.2410	0.4946	0.066*	
C11	0.3695 (7)	0.0987 (8)	0.4326 (3)	0.0499 (18)	
H11A	0.4665	0.1542	0.4362	0.060*	
H11B	0.3950	−0.0062	0.4333	0.060*	
C12	0.2901 (6)	0.1360 (6)	0.3664 (2)	0.0339 (15)	
H12A	0.2723	0.2434	0.3659	0.041*	
N1	−0.0014 (5)	0.0998 (6)	0.1591 (2)	0.0377 (13)	
N2	0.0625 (4)	0.1095 (6)	0.2950 (2)	0.0313 (12)	
H2B	0.0265	0.2041	0.2997	0.038*	
N3	0.3872 (5)	0.0999 (6)	0.3071 (2)	0.0366 (12)	
H3B	0.4298	0.0093	0.3132	0.044*	
H3C	0.4659	0.1662	0.3040	0.044*	

### Atomic displacement parameters (Å^2^)

**Table:**

	*U*^11^	*U*^22^	*U*^33^	*U*^12^	*U*^13^	*U*^23^
Cd1	0.0323 (2)	0.0391 (3)	0.02707 (19)	−0.0016 (3)	0.0035 (2)	0.0018 (2)
Br1	0.0462 (4)	0.0380 (4)	0.0405 (3)	0.0053 (3)	−0.0043 (3)	0.0000 (3)
Br2	0.0478 (4)	0.0428 (4)	0.0418 (3)	−0.0031 (4)	0.0129 (3)	0.0066 (3)
C1	0.056 (5)	0.061 (6)	0.039 (4)	0.011 (4)	−0.004 (3)	−0.001 (4)
C2	0.070 (5)	0.064 (6)	0.053 (4)	0.018 (5)	−0.020 (4)	−0.008 (4)
C3	0.047 (5)	0.070 (6)	0.076 (5)	0.013 (4)	−0.023 (4)	−0.036 (5)
C4	0.036 (4)	0.052 (4)	0.061 (4)	0.001 (4)	0.001 (4)	−0.020 (3)
C5	0.029 (4)	0.031 (4)	0.046 (4)	0.006 (3)	0.002 (3)	−0.011 (3)
C6	0.034 (4)	0.039 (4)	0.039 (4)	−0.005 (3)	0.006 (3)	−0.004 (3)
C7	0.036 (4)	0.033 (4)	0.032 (3)	−0.004 (3)	0.004 (3)	0.002 (3)
C8	0.051 (4)	0.048 (5)	0.035 (3)	−0.002 (4)	0.010 (3)	−0.004 (4)
C9	0.067 (5)	0.053 (5)	0.032 (4)	−0.004 (4)	0.017 (4)	0.000 (3)
C10	0.072 (5)	0.064 (5)	0.029 (3)	−0.001 (5)	−0.008 (4)	−0.005 (3)
C11	0.051 (4)	0.059 (5)	0.040 (4)	−0.001 (4)	−0.002 (3)	0.001 (4)
C12	0.041 (4)	0.033 (4)	0.028 (3)	−0.001 (3)	0.005 (3)	−0.009 (3)
N1	0.041 (3)	0.039 (3)	0.034 (3)	0.010 (3)	0.001 (2)	−0.003 (3)
N2	0.032 (3)	0.029 (3)	0.032 (3)	−0.006 (2)	−0.001 (2)	−0.003 (3)
N3	0.034 (3)	0.043 (3)	0.032 (3)	−0.005 (3)	0.001 (2)	−0.003 (3)

### Geometric parameters (Å, °)

**Table:**

Cd1—N3	2.313 (4)	C7—C12	1.520 (7)
Cd1—N2	2.366 (4)	C7—C8	1.532 (7)
Cd1—N1	2.373 (5)	C7—H7A	1.0000
Cd1—Br2	2.5621 (8)	C8—C9	1.522 (7)
Cd1—Br1	2.6054 (9)	C8—H8A	0.9900
C1—N1	1.342 (7)	C8—H8B	0.9900
C1—C2	1.372 (8)	C9—C10	1.506 (8)
C1—H1A	0.9500	C9—H9A	0.9900
C2—C3	1.370 (9)	C9—H9B	0.9900
C2—H2A	0.9500	C10—C11	1.526 (7)
C3—C4	1.377 (8)	C10—H10A	0.9900
C3—H3A	0.9500	C10—H10B	0.9900
C4—C5	1.383 (8)	C11—C12	1.520 (7)
C4—H4A	0.9500	C11—H11A	0.9900
C5—N1	1.338 (7)	C11—H11B	0.9900
C5—C6	1.502 (7)	C12—N3	1.483 (6)
C6—N2	1.468 (7)	C12—H12A	1.0000
C6—H6A	0.9900	N2—H2B	0.9300
C6—H6B	0.9900	N3—H3B	0.9200
C7—N2	1.483 (6)	N3—H3C	0.9200
			
N3—Cd1—N2	74.77 (15)	C7—C8—H8B	109.0
N3—Cd1—N1	145.44 (16)	H8A—C8—H8B	107.8
N2—Cd1—N1	70.73 (15)	C10—C9—C8	111.6 (5)
N3—Cd1—Br2	108.26 (12)	C10—C9—H9A	109.3
N2—Cd1—Br2	132.07 (13)	C8—C9—H9A	109.3
N1—Cd1—Br2	96.34 (13)	C10—C9—H9B	109.3
N3—Cd1—Br1	94.77 (13)	C8—C9—H9B	109.3
N2—Cd1—Br1	105.92 (12)	H9A—C9—H9B	108.0
N1—Cd1—Br1	92.79 (13)	C9—C10—C11	111.0 (5)
Br2—Cd1—Br1	121.01 (3)	C9—C10—H10A	109.4
N1—C1—C2	123.2 (7)	C11—C10—H10A	109.4
N1—C1—H1A	118.4	C9—C10—H10B	109.4
C2—C1—H1A	118.4	C11—C10—H10B	109.4
C3—C2—C1	117.6 (6)	H10A—C10—H10B	108.0
C3—C2—H2A	121.2	C12—C11—C10	110.9 (5)
C1—C2—H2A	121.2	C12—C11—H11A	109.5
C2—C3—C4	120.5 (6)	C10—C11—H11A	109.5
C2—C3—H3A	119.8	C12—C11—H11B	109.5
C4—C3—H3A	119.8	C10—C11—H11B	109.5
C3—C4—C5	118.7 (6)	H11A—C11—H11B	108.1
C3—C4—H4A	120.7	N3—C12—C11	111.7 (4)
C5—C4—H4A	120.7	N3—C12—C7	109.5 (4)
N1—C5—C4	121.4 (6)	C11—C12—C7	112.7 (5)
N1—C5—C6	116.4 (5)	N3—C12—H12A	107.6
C4—C5—C6	122.2 (6)	C11—C12—H12A	107.6
N2—C6—C5	111.4 (5)	C7—C12—H12A	107.6
N2—C6—H6A	109.3	C5—N1—C1	118.7 (5)
C5—C6—H6A	109.3	C5—N1—Cd1	114.3 (4)
N2—C6—H6B	109.3	C1—N1—Cd1	126.8 (4)
C5—C6—H6B	109.3	C6—N2—C7	114.4 (4)
H6A—C6—H6B	108.0	C6—N2—Cd1	105.1 (3)
N2—C7—C12	109.1 (4)	C7—N2—Cd1	109.1 (3)
N2—C7—C8	113.1 (4)	C6—N2—H2B	109.4
C12—C7—C8	110.9 (5)	C7—N2—H2B	109.4
N2—C7—H7A	107.8	Cd1—N2—H2B	109.4
C12—C7—H7A	107.8	C12—N3—Cd1	111.8 (3)
C8—C7—H7A	107.8	C12—N3—H3B	109.3
C9—C8—C7	112.9 (5)	Cd1—N3—H3B	109.3
C9—C8—H8A	109.0	C12—N3—H3C	109.3
C7—C8—H8A	109.0	Cd1—N3—H3C	109.3
C9—C8—H8B	109.0	H3B—N3—H3C	107.9

### Hydrogen-bond geometry (Å, °)

**Table:**

*D*—H···*A*	*D*—H	H···*A*	*D*···*A*	*D*—H···*A*
N3—H3B···Br2^i^	0.92	2.89	3.750 (5)	156
N3—H3C···Br1^ii^	0.92	2.59	3.498 (5)	168

Symmetry codes: (i) −*x*+1, *y*−1/2, −*z*+1/2; (ii) −*x*+1, *y*+1/2, −*z*+1/2.
